# Arbovirus emergence in the temperate city of Córdoba, Argentina, 2009–2018

**DOI:** 10.1038/s41597-019-0295-z

**Published:** 2019-11-21

**Authors:** Michael A. Robert, Daniela T. Tinunin, Elisabet M. Benitez, Francisco F. Ludueña-Almeida, Moory Romero, Anna M. Stewart-Ibarra, Elizabet L. Estallo

**Affiliations:** 1 Department of Mathematics, Physics, and Statistics, University of the Sciences, Philadelphia, PA 19104 USA; 2 Instituto de Investigaciones Biológicas y Tecnológicas (IIBYT) CONICET- Universidad Nacional de Córdoba. Centro de Investigaciones Entomológicas de Córdoba. Facultad de Ciencias Exactas, Físicas y Naturales, Universidad Nacional de Córdoba. Av. Vélez Sarsfield 1611. CP (X5016GCA). Ciudad Universitaria, Córdoba Capital, Argentina; 3 Cátedra de Matemática (Cs. Biológicas). Facultad de Ciencias Exactas, Físicas y Naturales, Universidad Nacional de Córdoba. Av. Vélez Sarsfield 1611. CP (X5016GCA). Ciudad Universitaria, Córdoba Capital, Argentina; 4 Department of Environmental Studies, State University of New York (SUNY) College of Environmental Science and Forestry, Syracuse, NY 13201 USA; 5 Department of Medicine, SUNY Upstate Medical University, Syracuse, NY 13210 USA; 6 Inter-American Institute for Global Change Research (IAI), Montevideo, Department of Montevideo Uruguay

**Keywords:** Infectious diseases, Epidemiology

## Abstract

The distribution of arbovirus disease transmission is expanding from the tropics and subtropics into temperate regions worldwide. The temperate city of Córdoba, Argentina has been experiencing the emergence of dengue virus, transmitted by the mosquito *Aedes aegypti*, since 2009, when autochthonous transmission of the virus was first recorded in the city. The aim of this work is to characterize the emergence of dengue and related arboviruses (Zika and chikungunya) in Córdoba since 2009. Herein, we present a data set with all known information about transmission of dengue, Zika, and chikungunya viruses in Córdoba, Argentina from 2009–2018, including what information is known of dengue virus (DENV) serotypes in circulation and origins of imported cases. The data presented in this work will assist researchers in investigating drivers of arbovirus emergence and transmission in Córdoba, Argentina and contribute to a better understanding of the global problem of the expanding distribution of arbovirus disease transmission.

## Abstract (summary)

**Table:** 

Measurement(s)	viral disease
Technology Type(s)	digital curation
Factor Type(s)	arbovirus • year
Sample Characteristic - Organism	Zika virus • Dengue virus group • Chikungunya virus • Dengue virus
Sample Characteristic - Location	Cordoba

Machine-accessible metadata file describing the reported data: 10.6084/m9.figshare.10120445

## Background & Summary

Dengue fever re-emerged in Latin America and the Caribbean in the 1980s, following the decline of widespread *Aedes aegypti* mosquito control programs aimed at eliminating Yellow Fever in prior decades^1^. Dengue fever is caused by dengue virus (DENV serotypes 1–4), which causes a spectrum of acute febrile illness^1^. In Argentina, dengue was reported for the first time in over 80 years in the northwestern Province of Salta in 1997, and has since been largely constrained to northern provinces of the country that have a subtropical climate^2,3^.

**Fig. 1:**
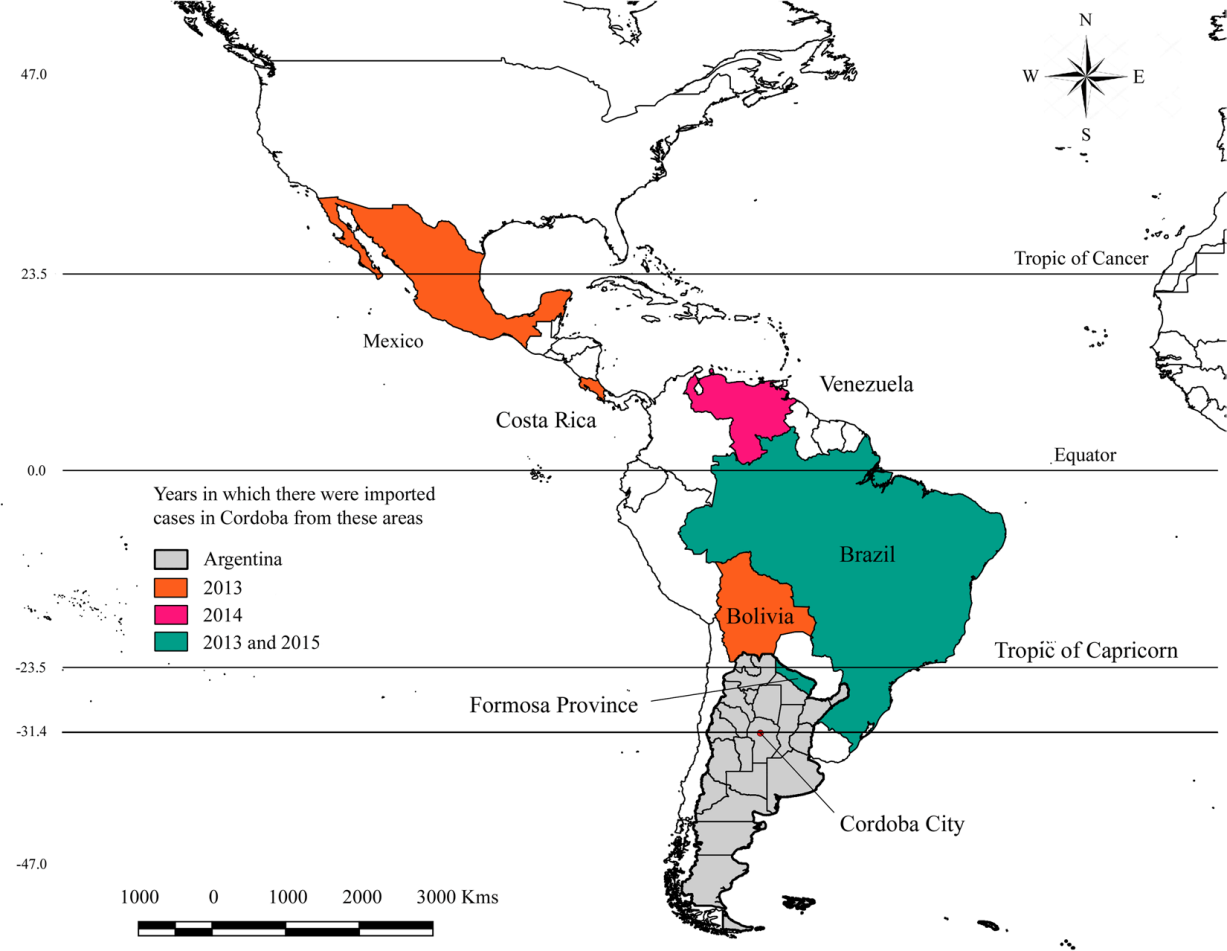
Location of Córdoba city within the province of Córdoba in Argentina. The orange, pink, and green highlighted countries are countries from which at least one dengue case was known to have been imported. Origins of imports were unknown for the majority of dengue cases reported. Lines of latitude are shown to emphasize the location of Córdoba city in relationship to the tropics.

Within the last decade, dengue emerged for the first time in areas with temperate climates, including Córdoba, the second largest city in Argentina (population 1.3 million), located in the southern cone of South America (Fig. 1). The first dengue outbreak in Córdoba occurred in 2009^4^, fourteen years after *Ae*. *aegypti* was first detected in the city^5^. Since that time, Córdoba has reported imported and autochthonous cases of *Aedes*-transmitted arboviruses most years; however, no prior studies have characterized the local epidemiological situation. Córdoba (31.4°S, 64.2°W) is among the southernmost cities in the Western Hemisphere to report autochthonous dengue transmission, making it an important site to study the dynamics of the recent emergence of arboviruses in southern temperate latitudes. Córdoba also reported imported cases of chikungunya and Zika virus in 2014 and 2016, respectively, as well as at least one case of autochthonous Zika virus in 2016^6^. Both chikungunya and Zika virus are transmitted primarily by the same vector as dengue virus.

Córdoba has a continental temperate climate, with warm summers (October – May, mean max temp = 25.1 °C) and dry, cool winters (June – September, mean min temp = 12.5 °C). Summer temperatures in Córdoba fall within the lower range of optimal temperatures for arbovirus transmission by *Ae*. *aegypti* (optimum peak: 28.5 °C, range: 13.5 °C–34.2 °C)^7^. The Córdoba province Ministry of Health has reported no vector activity during winter months; therefore, it is hypothesized that local outbreaks originate from imported arbovirus cases, often in travelers from arbovirus endemic areas.

In response to the emergence of dengue fever, the Ministry of Health (MoH) of Argentina together with the Córdoba Entomological Research Centre from the National University of Córdoba and the Institute of Biological and Technological Research (IIBYT) have increased efforts to control vectors. Vector control by the MoH is mostly focal, around homes with dengue cases, and includes control of adult mosquito populations (indoor and outdoor fumigation) and larval mosquitoes (*Bacillus thuringiensis israelensis* larvicide application, elimination of larval habitat)^8^. However, these efforts were unable to prevent outbreaks (as in 2016), and risk perception by the public remains low. Further investigation of the role of social and environmental drivers of arbovirus emergence in Córdoba is needed to develop effective vector control and disease management programs to reduce the burden of dengue illness.

The aim of this work was to present the epidemiological characteristics of the emergence of dengue fever and other arboviral diseases (i.e., chikungunya and Zika fever) in Córdoba over the last decade. We obtained epidemiological data by manually extracting records from reports published by the Argentinian National MoH (today the Argentinian National MoH is known as the Health Secretary due to National government decisions to change the category of Minister to the lower rank of Secretary)^6^. The availability of the data presented herein is critical to researchers interested in understanding patterns of emerging dengue transmission and, when complemented with other social-ecological data and epidemiological datasets from other sites would allow for the investigation of the many possible drivers of novel arbovirus emergence in temperate regions. Understanding the role of environmental, meteorological, and anthropogenic processes underlying the ongoing emergence of dengue in temperature regions may provide key insights to arbovirus emergence events occurring today and in the future under climate change at the range limits of arboviral disease across the globe^9^. Furthermore, by developing a better understanding of dengue emergence in temperate regions, researchers may be able to develop decision support tools for the public health sector that predict and mitigate future transmission and emergence events in similar regions worldwide.

## Methods

The data presented here were collected from public health reports provided regularly by the Argentinian National MoH^6^. The reports were published in Spanish and provided on a weekly basis in most years. We identified key data in the reports for characterizing arbovirus emergence, including the year-to-date cumulative number of probable (i.e., clinically diagnosed) and laboratory confirmed autochthonous (i.e., locally transmitted) and imported (i.e., illness in someone with travel history to a region with arbovirus activity) cases of dengue, Zika, and chikungunya virus in Argentina. In some years the reports included information on serotypes of dengue cases and/or regions associated with imported cases (i.e., regions where imported travelers were thought to have acquired the infection). In Córdoba, epidemiological and vector surveillance are the responsibilities of the Epidemiology Area of the Zoonosis Program of the MoH of the province (https://www.cba.gov.ar/epidemiologiaweb/). Suspected dengue infections that are diagnosed at either private or public clinical sites are reported to the National Health Surveillance System operated by the MoH. A subset of clinically diagnosed cases are confirmed by laboratory diagnostics (polymerase chain reaction (PCR) and immunoglobulin M antibodies to DENV) at the province of Córdoba Central Laboratories (Laboratorio Central de la Provincia de Córdoba). A subset of samples is sent to the Maiztegui Institute in Buenos Aires for confirmation using the plaque reduction neutralization test (PRNT). The same procedure is followed for all arbovirus cases (e.g. Zika and chikungunya cases).

We aimed to collect all available data on arbovirus cases including probable and laboratory confirmed cases, autochthonous and imported cases, DENV serotypes, and origins of imported cases. However, this information was not available in all reports. We translated, compiled, and reviewed the data to determine the weekly number of cases, source of imported cases, and DENV serotypes in circulation. We consolidated all reported cases of dengue, Zika, and chikungunya for Córdoba city between January 2009 and December 2018 (135 weekly reports). We cleaned the data to correct inconsistencies in reporting: we eliminated cases that appeared to be counted multiple times, for example when a single case appeared to be reported as both probable and confirmed and sums of probable and confirmed cases did not equal sums of total cases. The cases presented in this database are a sum of probable and confirmed cases. In Córdoba, all unconfirmed cases presenting as caused by an arbovirus were assumed by the MoH to be a specific arbovirus (e.g. dengue) given their proximity to other confirmed cases. Between 2009 and 2018, approximately 94.2% of all dengue cases were laboratory confirmed. Additionally, 52.9% of chikungunya cases and 75% of Zika virus cases were laboratory confirmed.

**Fig. 2:**
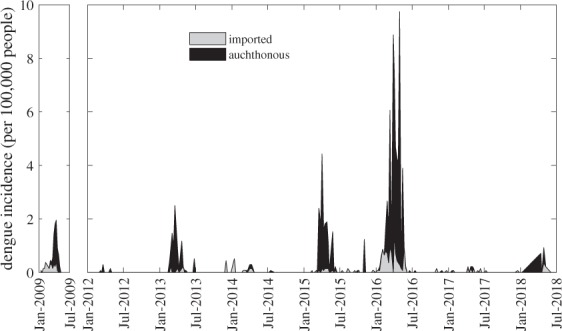
Incidence of imported (gray) and autochthonous (black) dengue cases relative to total incidence each epidemic week between January 2009–July 2018. Incidence is calculated as the number of cases per 100,000 inhabitants of Córdoba. Note: there was no reported dengue activity in 2010–2011, so this period has been excluded from the figure.

We calculated the incidence of imported and autochthonous cases each week (Fig. 2). Incidence is calculated as the number of cases per 100,000 inhabitants of Córdoba, where yearly population was estimated using census data from the most recent years with available population data (2001, 2008, and 2010) and linear interpolation^10,11^ (https://www.indec.gob.ar/indec/web/Nivel3-Tema-2-41). The population size in each year, *t*, was estimated by the function

$${\rm{P(t)\; =\; 8045}}{\rm{.47t}}+1257194.75,$$

where t = 0 is 2001.

No custom code was created in the development of this database.

## Data Records

The database is publicly available online (via figshare^12^) as a set of three comma separated files with weekly time series data for dengue, chikungunya, and Zika cases and one comma separated file with summary data for dengue serotypes and origins of imported cases. Data for each file begins in the year that the virus was first detected in Córdoba (2009, 2014, and 2016, respectively). The column headings of the files with weekly time series data are as follows.

YEAR: The year of the date of the entry.

DATE: The date of the MoH bulletin for the entry.

EW: The epidemiological week described within the MoH bulletin of the given date.

AUTO_CASES: The number of newly reported autochthonous cases (cases reported without history of recent travel outside of the country)

IMP_CASES: The number of newly reported imported cases (cases reported with history of recent travel outside of the country)

POP_SIZE: The population size of Córdoba in the YEAR of the entry (estimation method described in Methods)

AUTO_INCIDENCE: The incidence (per 100000 people) of newly reported autochthonous cases.

IMP_INCIDENCE: The incidence (per 100000 people) of newly reported imported cases.

In the fourth file describing dengue case serotypes and origins of imported cases, the following column headings are used.

YEAR: The year associated with the data presented.

N: The total number of cases tested for serotypes (NA indicates the number tested was unknown or not reported).

DENV: The total number of cases that were determined to be of serotype 1, 2, 3, or 4 as indicated by the column head.

ORIGINS_GLOBAL: The known origins of imported cases that were contracted outside of Argentina.

ORIGINS_ARGENTINA: The known origins of imported cases that were contracted in an Argentinian province other than Córdoba.

In Fig. 2, we present weekly incidence of cases from January 2009 to June 2018. A total of 1,429 dengue cases were reported during this period (1,170 autochthonous, 259 imported). DENV1 was the predominant serotype in circulation over the last decade, and DENV4 played a secondary role, although all four DENV serotypes were detected (Table 1). Imported dengue cases originated from tropical countries where dengue fever is endemic, as well as the endemic subtropical northern region of Argentina.

**Table 1:** Dengue virus serotypes detected in autochthonous and imported cases and the origin of imported dengue cases.

Year	DENV Serotypes Detected	Origins of Imported Cases
2009	DENV-1 (Majority; N unknown)	No Information
2010	No Information	
2011	No Information	
2012	DENV-1 (50%), DENV-4 (50%); (N = 2)	No Information
2013	DENV-1 (73.8%), DENV-2 (1.6%), DENV-3 (3.3%), DENV-4 (21.3%); (N = 61)	Brazil, Bolivia, Mexico, Costa Rica, Formosa Province (Argentina)
2014	DENV-1 (100%); (N = 2)	Venezuela
2015	DENV-1, DENV-4 (Frequency Unknown)	Brazil, Formosa Province (Argentina)
2016	DENV-1 (98.6%), DENV-2 (0.4%), DENV-3 (0.4%), DENV-4 (1.0%); (N = 288).	No Information
2017	No Information	
2018	DENV-1 (Majority; N Unknown)	Colombia

N is the number of cases in Córdoba that were tested for serotype each year. Note that in the serotype data, cases tested for serotype were not distinguished as to whether they were autochthonous or imported.

The first imported dengue case in Córdoba was reported Epidemiological Week (EW) 2 in 2009. In EW10, 2 cases of dengue in patients without travel to affected areas were confirmed, marking the first known autochthonous dengue case in Córdoba. In total, 88 autochthonous cases were reported between EW10-EW18. During EW2-EW18, 42 imported dengue cases were reported, leading to 130 total cases. All tested cases were confirmed to be DENV1^4^. The total dengue incidence in 2009 was 9.78 cases per 100,000 people.

In 2013, Córdoba experienced its second major outbreak, with 115 autochthonous and 10 imported cases reported (total incidence 10.13). Autochthonous and imported cases were reported from EW7-18. The known origins of imported cases include Brazil, Bolivia, Mexico, Costa Rica, and Formosa Province in northeastern Argentina.

In 2015, Córdoba experienced its third significant outbreak of dengue beginning with imported cases in EW5. From EW9-22, 221 autochthonous cases were reported. In total, 236 autochthonous and 14 imported dengue cases were reported (total incidence 19.01). DENV1 and DENV4 were detected, and cases were imported from Brazil and Formosa Province, Argentina.

Córdoba’s largest dengue outbreak to date began in EW52 of 2015 with an imported case of unknown origin. The first 2 autochthonous cases were reported in EW2, and 687 autochthonous and 134 imported cases were reported from EW2-24. In total, 822 cases of dengue were reported (688 autochthonous; 134 imported; total incidence 60.25). Of these, 288 (35%) cases were tested for DENV serotypes (Table 1). DENV2, DENV3, and DENV4 serotypes were detected in imported cases. Of the 284 DENV1 cases, 221 (78%) were autochthonous, and 63 (22%) were imported.

Chikungunya and Zika first emerged in Córdoba in 2014 and 2016, respectively (Table 2). A total of 23 imported chikungunya cases were reported from 2014 to 2017. In 2016, 9 imported cases and 1 autochthonous case of Zika acquired via sexual contact were reported. No cases of congenital Zika syndrome were detected.

**Table 2:** Imported and autochthonous chikungunya and Zika virus cases in Córdoba. No cases of chikungunya or Zika were reported prior to 2014.

Year	Chikungunya		Zika virus	
	Imported	Autochthonous	Imported	Autochthonous
2014	8	0	0	0
2015	6	0	0	0
2016	6	0	9	1
2017	3	0	0	0
2018	0	0	0	0

## Technical Validation

All data presented within this work is supported by reports published by the Argentinian Ministry of Health^6^. Previous work similarly used a combination of reported probable and confirmed cases to analyze outbreaks in the city of Córdoba in 2009^4^ and 2016^13^, and those data are consistent with the 2009 and 2016 data presented here.

## Usage Notes

The data presented herein characterizes the emergence of dengue in the temperate city of Córdoba, Argentina, and when coupled with complimentary social-ecological datasets, will facilitate the further investigation of mechanisms that are driving this ongoing emergence, including potential links with climate, dengue serotype circulation, and global travel. Before 2009, Córdoba was considered to be a population entirely susceptible to dengue. The data from outbreaks of dengue occurring in 2009, 2013, 2015, and 2016 provide a unique opportunity to estimate the basic reproduction number (R_0_) of dengue in Córdoba, which will be helpful to investigate potential impacts of vector control measures and eventual vaccine deployment in the city through mathematical modeling studies. Although complete serotype information on dengue cases is lacking in the present database, the information provided on the serotypes in circulation (i.e. from both autochthonous and imported cases) can be important for predicting outbreaks^14^. The overwhelming presence of DENV1 in outbreaks for which serotype information is available should give public health officials reason to take extra precautions in the event that transmission of other dengue serotypes occurs given the severe symptoms known to occur in secondary dengue infections^1^. Furthermore, the presentation of this data highlights that detailed clinical epidemiological data on dengue transmission is lacking and that future studies of arbovirus transmission in Córdoba should consider investigating serotype-specific seroprevalence across age groups (e.g via prospective serosurveys) and travel history of seropositive individuals.

In the last decade, *Ae*.-transmitted arbovirus-related illness has emerged for the first time in Córdoba. The city has quickly become a site of significant epidemic dengue transmission, as indicated by the occurrence of 4 outbreaks in less than 10 years, and local transmission most years since 2009. During outbreaks, cases peaked during the latter part of the summer, when vector densities were elevated and maximum daily temperatures were slightly lower than the optimum temperature for arbovirus transmission by *Ae*. *aegypti* of 28.5 °C^7^. The increase in arbovirus activity in Córdoba mirrors that of arbovirus activity across temperate regions of the world, including the southern United States and southern Europe^15^. Autochthonous cases of dengue re-emerged after many decades of no transmission in Florida, USA, in 2009^16^, and in France, Croatia, and Portugal in 2010–2013^17^. Increasing arbovirus transmission in temperate latitudes is likely associated with social and ecological factors including greater human movement, expansion and local adaptation of *Ae*. mosquitoes, and changes in climate resulting in increased surface temperatures and altered rainfall patterns^18,19^. The data presented herein, complemented with local vector and climate data (i.e., temperature, precipitation, climate), will be useful for estimating links between meteorological patterns and anomalies and dengue transmission, which could provide useful insights not only for Córdoba, but for temperate regions across the globe.

As Córdoba is a temperate region, local transmission depends on the importation of cases from dengue endemic areas. Much of this importation is suspected to occur currently from travelers returning from northern provinces of Argentina and neighboring countries such as Brazil, Bolivia, and Paraguay after holidays in the summer and autumn months (e.g., Christmas, Carnival, and Easter). With the current political crisis in Venezuela, resulting in mass migration of people into Argentina, the risk of importation of dengue fever and other mosquito borne diseases (e.g., malaria) has increased^20^. Regional efforts are needed to strengthen surveillance of arbovirus transmission, due to the key role of human movement in dengue introductions. The data presented in this work combined with arbovirus transmission data from other parts of Argentina as well as other countries in South and Central America, together with human movement data (e.g., airline records, bus/train records, etc.) could provide insights into the role that human movement has played in the ongoing emergence of dengue in Córdoba and provide guidance for preventing future outbreaks. The present study is focused on providing a detailed description of the arbovirus transmission data available for Córdoba in order to highlight in the recent and ongoing emergence of dengue in the city. Given the history of dengue importations from northern provinces of Argentina into Córdoba, an accessible nationwide database of arbovirus cases in Argentina would be critical for understanding patterns of transmission that lead to localized outbreaks in cities such as Córdoba (Table 1).
